# Thiopental and decompressive craniectomy as last-tier ICP-treatments in aneurysmal subarachnoid hemorrhage: is functional recovery within reach?

**DOI:** 10.1007/s10143-023-02138-6

**Published:** 2023-09-07

**Authors:** Sofie Björk, Anders Hånell, Elisabeth Ronne-Engström, Anton Stenwall, Fartein Velle, Anders Lewén, Per Enblad, Teodor Svedung Wettervik

**Affiliations:** Department of Medical Sciences, Section of Neurosurgery, Uppsala University, 751 85 Uppsala, Sweden

**Keywords:** Aneurysmal subarachnoid hemorrhage, Decompressive craniectomy, Intracranial pressure, Outcome, Thiopental

## Abstract

The study aimed to investigate the indication and functional outcome after barbiturates and decompressive craniectomy (DC) as last-tier treatments for elevated intracranial pressure (ICP) in aneurysmal subarachnoid hemorrhage (aSAH). This observational study included 891 aSAH patients treated at a single center between 2008 and 2018. Data on demography, admission status, radiology, ICP, clinical course, and outcome 1-year post-ictus were collected. Patients treated with thiopental (barbiturate) and DC were the main target group.

Thirty-nine patients (4%) were treated with thiopental alone and 52 (6%) with DC. These patients were younger and had a worse neurological status than those who did not require these treatments. Before thiopental, the median midline shift was 0 mm, whereas basal cisterns were compressed/obliterated in 66%. The median percentage of monitoring time with ICP > 20 mmHg immediately before treatment was 38%, which did not improve after 6 h of infusion. Before DC, the median midline shift was 10 mm, and the median percentage of monitoring time with ICP > 20 mmHg before DC was 56%, which both significantly improved postoperatively. At follow-up, 52% of the patients not given thiopental or operated with DC reached favorable outcome, whereas this occurred in 10% of the thiopental and DC patients.

In summary, 10% of the aSAH cohort required thiopental, DC, or both. Thiopental and DC are important integrated last-tier treatment options, but careful patient selection is needed due to the risk of saving many patients into a state of suffering.

**Supplementary Information:**

The online version contains supplementary material available at 10.1007/s10143-023-02138-6.

## Introduction

Aneurysmal subarachnoid hemorrhage (aSAH) is a rare type of stroke that causes a high burden of mortality and morbidity [30]. General management is focused on early occlusion of the ruptured aneurysm to avoid rebleeding, drainage of cerebrospinal fluid (CSF) due to acute hydrocephalus, and prevention and management of delayed ischemic neurological deficits (DIND) [7, 10].

In addition, neurointensive care (NIC) management seeks to improve the cerebral environment through physiological optimization [6, 26, 31, 33, 36, 45, 48]. A main NIC target is to control intracranial pressure (ICP) to avoid brain herniation and maintain the cerebral perfusion pressure (CPP) at sufficiently high levels to prevent ischemia [36, 45]. Previous studies indicate that ICP elevation is common after aSAH [26, 31, 33, 48], both immediately after the primary bleeding and due to secondary causes (e.g., rebleeding, hydrocephalus, and vasogenic/cytotoxic brain edema) [33, 36]. In unconscious patients, ICP is typically monitored with an external ventricular drainage (EVD) system [36]. Elevated ICP can often be avoided using CSF drainage, evacuation of a large intracerebral hemorrhage (ICH), and maintenance of pCO_2_ and electrolytes within normal ranges [7, 10, 36]. A subset of all aSAH patients, especially those with severe early brain injury and secondary infarctions, does not respond to basal treatments and develops refractory intracranial hypertension [2, 3, 8, 11, 15, 20, 27, 42]. In these cases, last-tier treatments, including barbiturates or decompressive craniectomy (DC), can be used to control ICP. Barbiturates are powerful sedatives that induce metabolic suppression, reducing cerebral blood flow and cerebral blood volume and thereby decreasing ICP [28]. Barbiturates are recommended to control refractory intracranial hypertension in traumatic brain injury (TBI) [5]. They are also used for the same purposes in aSAH [33, 36]. However, there are few studies on the optimal indication for this treatment and its cerebral physiological effects in this disease [3]. DC has been studied in randomized controlled trials and meta-analyses in several other acute brain injuries [21, 24, 29]. Specifically, DC is currently used to alleviate intracranial hypertension in TBI [16, 24] and malignant middle cerebral artery infarction in brain herniation [21, 29, 41]. However, its use in aSAH is less clear and has chiefly been evaluated in a few cohort studies [2, 8, 11, 15, 19, 42]. In aSAH, DC has been used both as a primary procedure for patients who present with immediate brain herniation (often due to ICH) after ictus [4] or secondary DC [2, 8] due to delayed deterioration with elevated ICP. Approximately 10% of all aSAH patients are treated with DC. Yet, although it often reduces elevated ICP immediately, there are concerns that the patients remain in a poor neurological condition without a reasonable chance of functional recovery [2, 8, 11, 15, 19, 42].

This study assessed the indications for barbiturates/DC and outcome after these treatments in aSAH patients treated according to an escalated ICP-protocol at a single center. We hypothesized that these patients exhibited severe intracranial hypertension (based on radiological signs and ICP measurements), which would improve after treatment. However, few would achieve functional recovery due to the severity of the underlying brain injury.

## Materials and methods

### Patients and study design

In this retrospective, observational study, aSAH patients admitted between 2008 and 2018 to the Department of Neurosurgery at the University Hospital in Uppsala, Sweden, were eligible for inclusion. Of 913 aSAH patients, 22 were initially treated at another NIC unit and were excluded. Thus, the final study population included 891 aSAH patients.

### Treatment protocol

**Fig. 1:**
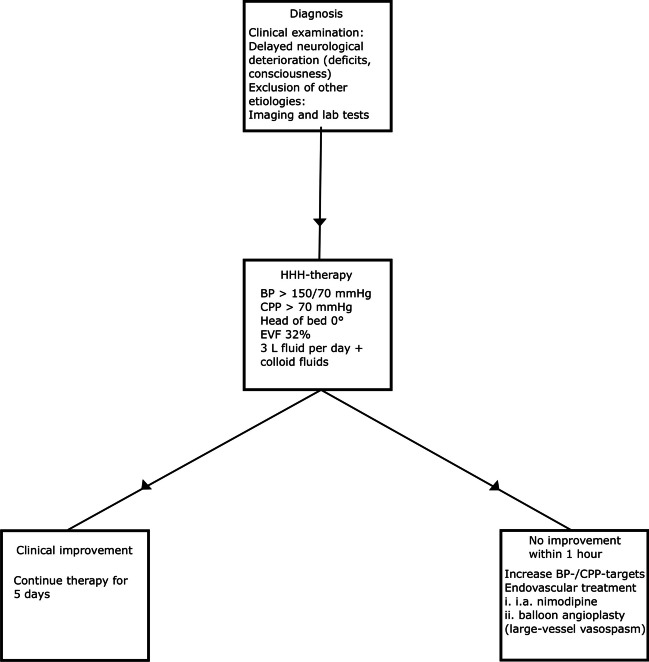
DIND diagnosis and treatment. The figure illustrates the diagnosis and treatment of DIND. BP, blood pressure; EVF, erythrocyte volume fraction

Patients with aSAH within our catchment area were admitted to our NIC or neurointermediate care unit. Previous studies have thoroughly described our clinical management protocol [31–44]. Treatment goals were ICP ≤ 20 mmHg, CPP ≥ 60 mmHg, pO_2_ ≥ 12 kPa, arterial glucose 5–10 mmol/L (mM), electrolytes within normal ranges, and body temperature < 38 °C. Aneurysms were treated as early as possible, by endovascular intervention or surgical clipping [22]. Nimodipine was administered to all patients after admission to our department. aSAH patients who were unconscious (Glasgow Coma Scale Motor score < 6) were mechanically ventilated and sedated. Neurological wake-up tests were performed six times per day for these patients. DIND was defined as a focal neurological deficit or deterioration in consciousness that occurred after some delay from ictus and was not explained by hydrocephalus, rebleeding, or meningitis. NIC variables (e.g., ICP and CPP) were not included in the assessment of DIND. If there were no manifest cerebral infarction on computed tomography (CT), HHH (hypertension, hypervolemia, and hemodilution)-therapy was initiated [12]. If the patient did not respond to this therapy, endovascular intervention with intra-arterial calcium-channel blockage was started and exceptionally also balloon angioplasty would be performed in case of radiological vasospasm (Fig. 1). CPP was maintained by intravenous fluids at first hand and vasopressors/inotropes at second hand. ICP was controlled according to an escalated protocol, as described below.

### ICP-treatments – step 1

**Fig. 2:**
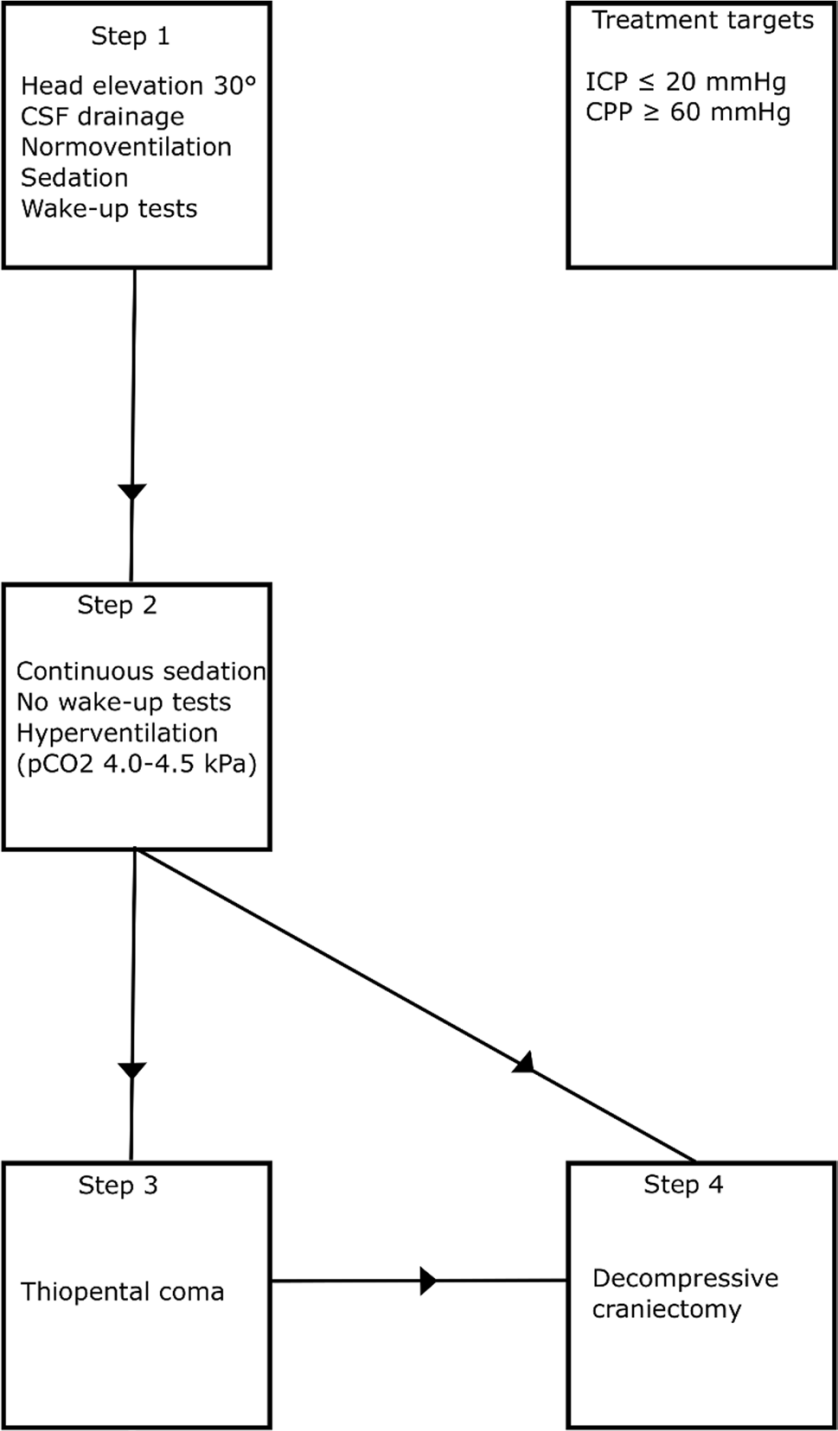
Escalated ICP-treatments. The figure illustrates the escalation of treatments to keep ICP below 20 mmHg. The specific steps are described in detail in the text

An EVD was inserted for ICP monitoring and possibly CSF drainage in unconscious patients with or without hydrocephalus (Fig. 2). The EVD was typically opened at 15 mmHg in the event of ICP elevation. When the ventricles were small and an EVD could not be placed, an intraparenchymal ICP monitor was inserted. The head of the patient bed was elevated to 30°. The patients were typically close to normoventilated with a pCO_2_ between 5.5 and 6.0 kPa.

### ICP-treatments – step 2

The patients were mildly hyperventilated (pCO_2_ between 4.0 and 4.5 kPa) and continuously sedated without wake-up tests.

### ICP-treatments – step 3

If steps 1 and 2 failed to control ICP, thiopental infusion was initiated (provided no significant midline shift). This included cases of persistent ICP elevation following rebleeding. The infusion was started with a bolus dose of 4–8 mg/kg until ICP decreased below 20 mmHg or CPP became too low. Thiopental was then continuously infused with 5–10 mg/kg/h for 6 h and then 2–5 mg/kg/h. The goal was not to achieve burst suppression, but to administer the lowest possible dose to keep ICP below 20 mmHg. When thiopental was given, a CPP of 50 mm Hg was sufficient. Thiopental concentrations in blood were assessed daily and levels above 380 μmol/L were avoided.

### ICP-treatments – step 4

Primary DC without ICP monitoring was done early in case of severe mass effect/perioperative brain swelling in association with surgical clipping (usually after the simultaneous evacuation of an ICH). Secondary DC was a last-tier treatment and performed if any of these four indications were met: (i) midline shift, but no significant ICH to evacuate (thiopental contraindicated), (ii) elevated ICP despite thiopental treatment, (iii) adverse effects of thiopental, and (iv) the patient was judged not to tolerate thiopental (patients with high risk of severe adverse events and multiple organ failure, e.g., older adults and patients with respiratory, circulatory, and kidney failure or more severe infections). However, DC was withheld for patients with extreme ICP elevation (above 50 mmHg) who did not respond to thiopental with severe brain injuries on imaging, when functional recovery was considered beyond reach. The DC of choice was hemi-DC if there was a lateralized brain injury/swelling with midline shift. A bifrontal DC was performed in the event of diffuse, bilateral brain swelling when no midline shift was present, sparing a bone ridge in the midline, if the patients did not tolerate or respond to thiopental infusion. In all cases, the aim was to maximize decompression by removing a large bone flap combined with duraplasty.

### Cranioplasty

Following DC, cranioplasty (CP) was scheduled 3 months after DC, but often occurred later due to long waiting lists [34]. Autologous bone flaps from the DC were saved and stored in a – 70 °C freezer until CP. For long waiting times for CP, a synthetic implant was used, based on the decision of the responsible neurosurgeon.

### Clinical, radiological, and physiological variables

Clinical variables (demography, admission status, and treatments) were extracted from medical records and the NIC database. The amount of bleeding on the initial CT scan was evaluated by Fisher grade [13] and according to the more granular Hijdra score [17, 23, 25]. The Hijdra score (max = 42) is the sum of the cistern score (grade 0 to 3 in 10 cisterns) and the ventricle score (grade 0 to 3 in 4 ventricles). These assessments were conducted by one of the authors (TSW). Rebleeding was defined as the combination of clinical deterioration and more SAH on the follow-up CT. Radiological signs of mass effect, including midline shift (mm), basal cisterns (open, compressed, obliterated), and convexity sulci (open, compressed, obliterated), were assessed on the CT immediately before and after thiopental infusion and DC surgery.

ICP was monitored with an external ventricular drainage system (EVD; HanniSet, Xtrans, Smith Medical GmbH, Glasbrunn, Germany) or an intraparenchymal sensor device (Codman ICP Micro-Sensor, Codman & Shurtleff, Raynham, MA, USA). Arterial blood pressure was measured invasively in the radial artery at heart level. Physiological data were collected at 100 Hz using the Odin software [18]. Median ICP and CPP were calculated during the last hour before DC and 1 h immediately after DC. The same variables were calculated in the previous hour before thiopental infusion and between the 5th and 6th hour after initiation of the infusion, when the effect of the treatment was expected to have stabilized. The percentage of monitoring time of ICP above 20 mmHg and CPP below 60 mmHg was also calculated for the same time windows. The ICP- and CPP-thresholds were chosen as per our treatment protocol.

### Outcome

Functional outcome was assessed 12 months post-ictus, using the Glasgow Outcome Scale- Extended (GOS-E) [37, 47]. Trained staff held structured telephone interviews with the patient or their next of kin. Functional outcome was dichotomized into favorable/unfavorable (GOS-E 5–8/1–4) and survival/mortality (GOS-E 2–8/1).

### Statistical analysis

Data were reported as numbers (proportion) or medians (interquartile range (IQR). Differences in demography, admission variables, hemorrhage severity, clinical course, treatments, and functional outcome among those with no last-tier (thiopental/DC) treatment, thiopental alone, and DC surgery (with and without prior thiopental treatment) were assessed with chi-square (with *z* test), McNemar Bowker, Mann–Whitney *U*-, or Kruskal–Wallis test, depending on the type of data. A *p* value < 0.05 was considered statistically significant. All statistical analyses were conducted using SPSS version 28 (IBM Corp, Armonk, NY, USA).

### Ethics

The study was approved by the Swedish Ethical Review Authority.

## Results

### Patient characteristics

In the entire cohort of 891 aSAH patients (Table 1), the median age was 59 (IQR 51–67) years and 67% were female. At admission, the median WFNS grade was 2 (IQR 1–4), 10% exhibited unreactive pupil(s), the median Fisher grade was 3 (IQR 3–4), and the median Hijdra score was 17 (IQR 9–25). The ruptured aneurysm was in the anterior circulation in 84% of patients. Seventy-two percent of the aneurysms were treated with embolization and 21% with clipping. Rebleeding occurred in 8% of patients. Eleven percent required ICH evacuation; and in this subgroup of patients, 84% were treated with clipping, and the ruptured aneurysm was on the middle cerebral artery (MCA) in 68% of these cases. Nineteen percent of all patients developed DIND. Ten percent developed post-hemorrhagic hydrocephalus and were treated with a ventriculo-peritoneal (VP)-shunt later. Ten percent died during NIC and 17% were deceased 1-year after ictus. Finally, 48% reached a favorable outcome 1-year post-ictus.

**Table 1:** Patient demographics, admission variables, clinical course, and outcome in thiopental and decompressive craniectomy treatment. The *p* values reflect the Kruskal–Wallis-/chi-square tests. Different superscripted letters (^a,b^) indicate a statistically significant difference between the groups in the post-hoc Dunn’s-/*z* test. Thirty-one patients had no available outcome data

	All	No thiopental/DC	Thiopental alone	DC	*p* value
Patients, *n* (%)	891 (100%)	800 (90%)	39 (4%)	52 (6%)	NA
Age (years), median (IQR)	59 (51–67)	60 (51–68)^a^	53 (44–59)^b^	54 (46–59)^b^	< 0.001
Sex (male/female), *n* (%)	291/600 (33/67%)	265/535 (33/67%)^a^	12/27 (31/69%)^a^	14/38 (27/73%)^a^	0.63
WFNS grade, median (IQR)	2 (1–4)	2 (1–4)^a^	4 (4–5)^b^	4 (4–5)^b^	< 0.001
Pupillary status (abnormal), *n* (%)	89 (10%)	69 (9%)^a^	9 (23%)^b^	11 (21%)^b^	< 0.001
Fisher, median (IQR)	3 (3–4)	3 (3–4)^a^	4 (3–4)^a^	4 (3–4)^b^	< 0.001
Hijdra score, median (IQR)	17 (9–25)	16 (9–25)^a^	22 (10–30)^a^	24 (17–29)^b^	< 0.001
Aneurysm location					
ICA, *n* (%)	199 (22%)	176 (22%)^a^	12 (31%)^a^	11 (21%)^a^	< 0.001
ACA, *n* (%)	353 (40%)	332 (42%)^a^	16 (41%)^a^	5 (10%)^b^	
MCA, *n* (%)	199 (22%)	159 (20%)^a^	4 (10%)^a^	36 (69%)^b^	
Posterior circulation, *n* (%)	140 (16%)	133 (17%)^a^	7 (18%)^a^	0 (0%)^b^	
Rebleeding *n* (%)	71 (8%)	50 (6%)^a^	8 (21%)^b^	13 (25%)^b^	< 0.001
Treatment					
No, *n* (%)	62 (7%)	56 (7%)^a^	5 (13%)^a^	1 (2%)^a^	< 0.001
Embolization, *n* (%)	638 (72%)	593 (74%)^a^	27 (69%)^a^	18 (35%)^b^	
Clipping, *n* (%)	184 (21%)	149 (19%)^a^	6 (15%)^a^	29 (56%)^b^	
Both, *n* (%)	7 (1%)	2 (0%)^a^	1 (3%)^a,b^	4 (8%)^b^	
Hematoma evacuation, *n* (%)	101 (11%)	60 (8%)^a^	5 (13%)^a^	36 (69%)^b^	< 0.001
ICP monitoring					
None, *n* (%)	337 (38%)	337 (42%)^a^	0 (0%)^b^	0 (0%)^b^	< 0.001
EVD, *n* (%)	500 (56%)	443 (55%)^a^	23 (59%)^a^	34 (65%)^a^	
Codman, *n* (%)	14 (2%)	9 (1%)^a^	2 (5%)^a,b^	3 (6%)^b^	
Both, *n* (%)	40 (4%)	11 (1%)^a^	14 (36%)^b^	15 (29%)^b^	
DIND, *n* (%)	169 (19%)	144 (18%)^a^	15 (38%)^b^	10 (19%)^a,b^	0.006
Shunt (yes), *n* (%)	91 (10%)	78 (10%)^a^	2 (5%)^a^	13 (25%)^b^	0.001
GOS-E*, median (IQR)	4 (3–6)	5 (3–6)^a^	1 (1–3)^b^	3 (2–3)^c^	< 0.001
Favorable outcome, *n* (%)	411 (48%)	402 (52%)^a^	4 (10%)^b^	5 (10%)^b^	< 0.001
Mortality, *n* (%)	149 (17%)	109 (14%)^a^	27 (69%)^b^	13 (25%)^a^	< 0.001
Mortality during NIC, *n* (%)	90 (10%)	58 (8%)^a^	25 (64%)^b^	7 (13%)^a^	< 0.001

### Trajectories to thiopental and decompressive craniectomy treatment

Thirty-nine (4%) patients were treated with thiopental alone and 52 (6%) with DC (Tables 1 and 2). In the thiopental group, treatment indication was ICP elevation directly associated with rebleeding in eight patients and later ICP elevations in 31. In the DC group, two cases were of primary type without prior ICP monitoring, 16 patients were done after thiopental treatment, and 34 after ICP monitoring without prior thiopental treatment. One primary DC was conducted due to perioperative brain swelling associated with surgical clipping, and the other due to early neurological deterioration combined with radiological signs of mass effect without prior ICP monitoring. All secondary DCs were performed due to radiological signs of mass effect and high ICP. One DC was bifrontal while the rest were hemicraniectomies.

**Table 2:** Pathways to last-tier treatments. Of 52 DC procedures, 1 was bifrontal and the remaining 51 were hemi-DCs. *Two numbers are given; the left describes the indication for thiopental and the right describes the indication for DC

Treatment	Total	Indication			
		Rebleeding	Perioperative swelling	Neurological deterioration plus radiology	ICP plus radiology
Thiopental alone, *n*	39	8	0	0	31
ICP- > Thiopental- > DC*, *n*	16	7/0	0/0	0/0	9/16
ICP—> DC, *n*	34	0	0	0	34
Primary DC, *n*	2	0	1	1	0
Total*, *n*	91	15/0	0/1	0/1	40/50

### Thiopental and decompressive craniectomy—demography, injury severity, and early clinical course

The treatment groups had some differences in demography, injury characteristics, and clinical courses (Table 1). The patients treated with thiopental alone or DC surgery were younger (*p* < 0.001), exhibited a higher WFNS grade (*p* < 0.001), and more often showed abnormal pupillary reactivity (*p* < 0.001) at admission than those who did not receive these treatments (Table 1). The DC patients also had more blood on the first CT with a higher Fisher and Hijdra score (*p* < 0.001) than the other two groups (Table 1). DC patients more frequently had their ruptured aneurysm on the MCA, but less often on the anterior cerebral artery (ACA) or the posterior circulation than the cohort with thiopental alone or no last-tier ICP treatment (*p* < 0.001, Table 1). The thiopental and DC groups exhibited a higher rebleeding rate than those without last-tier ICP treatment (*p* < 0.001). In contrast, the DC cohort had more often undergone surgery with ICH evacuation (*p* < 0.001). Thiopental alone patients more frequently showed DIND than the no thiopental/DC and the DC group (*p* = 0.006, Table 1). The DC group more often developed post-hemorrhagic hydrocephalus and was treated with a VP-shunt more frequently than the other two groups (*p* = 0.001).

### Thiopental and decompressive craniectomy—mass effect on imaging and intracranial pressure dynamics before and after treatment

Before thiopental (Table 3), the median midline shift was 0 (IQR 0–5) mm, whereas many patients had compressed/obliterated cisterns (66%) and compressed/obliterated convexity sulci (82%). On the follow-up CT, midline shift was unchanged, but more thiopental patients exhibited compressed/obliterated cisterns (79%) or compressed/obliterated convexity sulci (84%). The median percentage of monitoring time with ICP > 20 mmHg was 38% (IQR 5–90%), and the median percentage of monitoring time of CPP < 60 mmHg was 2% (IQR 0–20%) 1 h before thiopental infusion. These variables did not significantly change on the 6th hour following infusion start (Table 3). If the 25 thiopental cases who died (24 due to severe intracranial hypertension and 1 due to circulatory arrest) during NIC were excluded from these analyses, the radiological mass effect did not increase. The median percentage of monitoring time with ICP > 20 mmHg decreased from 29% (IQR 4–82) before thiopental to 0% (IQR 0–3) after thiopental infusion.

**Table 3:** Mass effect and cerebral physiology before and after thiopental. One of the patients treated with thiopental had no available radiological imaging pre- and postoperatively. ICP and CPP were analyzed 1 h before and between hours 5 and 6 in the group treated with thiopental infusion without DC

Radiological mass effect before and after thiopental						
Variables	Thiopental alone—all cases			Thiopental alone—NIC survivors		
	Before	After	p value	Before	After	p value
Midline shift (mm), median (IQR)	0 (0–5)	0 (0–5)	0.73	0 (0–2)	0 (0–2)	0.18
Basal cisterns (open/compressed/obliterated), *n* (%)	14/20/4 (37/53/11%)	8/18/12 (21/47/32%)	0.03	6/8/0 (43/57/0%)	6/8/0 (43/57/0%)	1.00
Convexity sulci (open/compressed/obliterated), *n* (%)	7/25/6 (18/66/16%)	6/16/16 (16/42/42%)	0.02	5/6/3 (36/43/21%)	5/6/3 (36/43/21%)	0.39
Cerebral physiology before and after thiopental						
Variables	Thiopental alone—all cases			Thiopental alone—NIC survivors		
	Before	After	p value	Before	After	p value
ICP (mmHg), median (IQR)	19 (12–24)	20 (11–54)	0.41	19 (12–22)	9 (5–18)	0.01
ICP > 20 mmHg (% monitoring time), median (IQR)	38 (5–90)	38 (7–83)	0.53	29 (4–82)	0 (0–3)	0.07
CPP (mmHg), median (IQR)	74 (65–85)	75 (46–82)	0.35	71 (64–79)	76 (65–82)	0.17
CPP < 60 mmHg (% monitoring time), median (IQR)	2 (0–20)	0 (0–100)	0.32	3 (0–16)	0 (0–0)	0.13

Before DC, the median midline shift was 10 (IQR 7–12) mm, and most patients had compressed/obliterated cisterns (90%) and compressed/obliterated convexity sulci (98%), as shown in Table 4. On the follow-up CT, the median midline shift was 2 (IQR 1–5) mm, 22% exhibited compressed/obliterated cisterns, and fewer (63%) patients had compressed/obliterated convexity sulci. Moreover, before DC, the median percentage of monitoring time of ICP > 20 mmHg was 56% (IQR 13–91%), and the median percentage of monitoring time of CPP < 60 mmHg was 5% (IQR 0–32%), which significantly improved to a median of 0% in both variables after DC (Table 4). The exclusion of seven patients (three with severe intracranial hypertension post-DC and four due to circulatory arrest after withdrawal of care) who died during NIC from these analyses did not impact the results.

**Table 4:** Mass effect and cerebral physiology before and after decompressive craniectomy. Three patients treated with DC had no available radiological imaging pre- and postoperatively. ICP and CPP were analyzed 1 h before and 1 h after DC

Radiological mass effect before and after DC						
Variables	DC–all cases			DC–NIC -survivors		
	Before	After	p value	Before	After	p value
Midline shift (mm), median (IQR)	10 (7–12)	2 (1–5)	< 0.001	10 (7–13)	2 (1–5)	< 0.001
Basal cisterns (open/compressed/obliterated), *n* (%)	5/22/22 (10/45/45%)	38/10/1 (78/20/2%)	< 0.001	4/21/18 (9/49/42%)	34/8/1 (79/19/2%)	< 0.001
Convexity sulci (open/compressed/obliterated), *n* (%)	1/28/20 (2/57/41%)	18/30/1 (37/61/2%)	< 0.001	1/24/18 (2/56/42)	17/25/1 (40/58/2%)	< 0.001
Cerebral physiology before and after DC						
Variables	DC – all cases			DC – NIC -survivors		
	Before	After	p value	Before	After	p value
ICP (mmHg), median (IQR)	21 (18–25)	6 (3–10)	< 0.001	20 (18–25)	6 (3–9)	< 0.001
ICP > 20 mmHg (% monitoring time), median (IQR)	56 (13–91)	0 (0–0)	< 0.001	47 (13–93)	0 (0–0)	< 0.001
CPP (mmHg), median (IQR)	67 (62–77)	80 (71–90)	< 0.001	68 (63–79)	80 (71–91)	< 0.001
CPP < 60 mmHg (% monitoring time), median (IQR)	5 (0–32)	0 (0–3)	< 0.001	3 (0–12)	0 (0–3)	0.002

### Decompressive craniectomy–cranioplasty outcomes

After DC, 31 (60%) patients underwent CP: one patient refused to proceed with CP, 17 died before CP, and three were lost to follow-up as they were discharged to another neurosurgical facility (Supplementary Table 1). CP was done in a median of 7 (IQR 4–9) months post-DC, and autologous bone rather than synthetic implants was used in almost all cases (94%). Of these 31 CP patients, 13 (42%) required a re-operation: 6 (20%) underwent surgical removal of the implant due to infection, 5 (16%) due to bone resorption, and in 2 (6%) implant re-positioning was performed.

### Thiopental and decompressive craniectomy—long-term clinical outcome

**Fig. 3:**
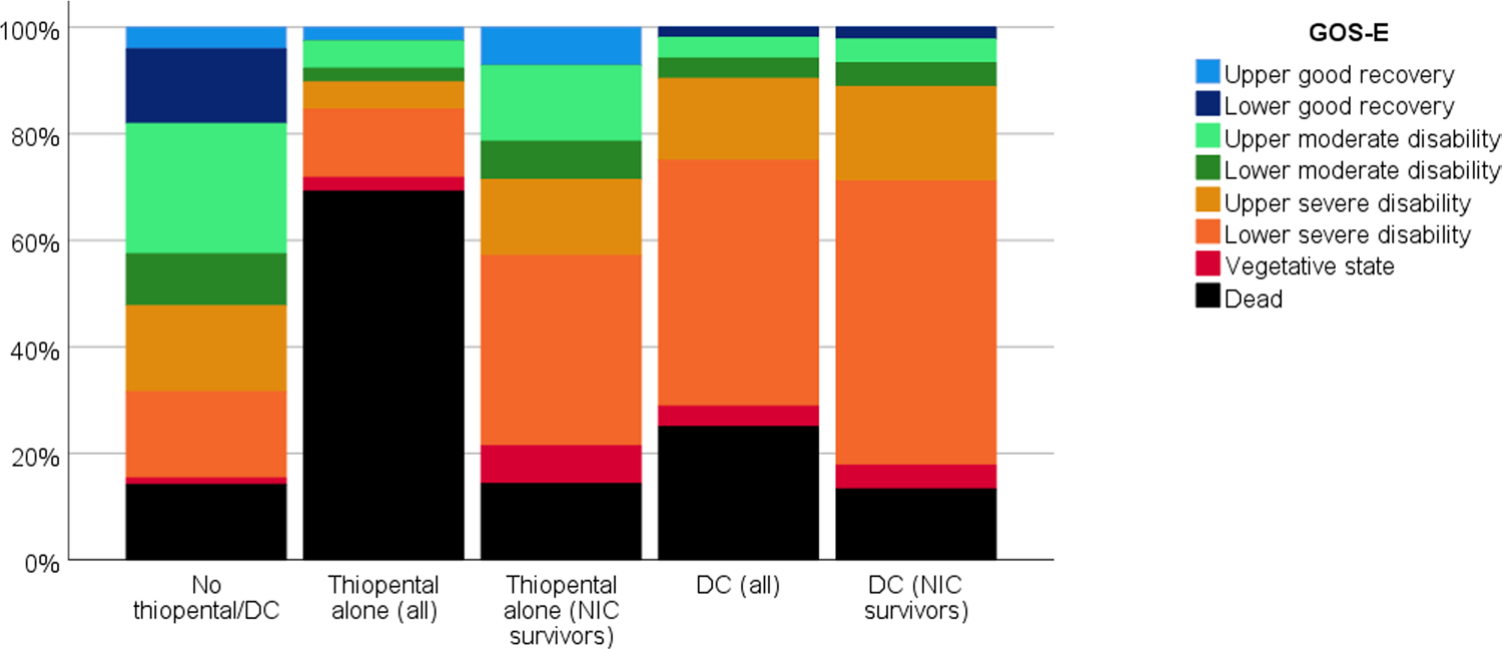
Clinical outcome at 1-year follow-up post-ictus

Thiopental and DC patients exhibited a lower rate of favorable outcome (both groups at 10%), compared to patients (48%) who did not receive these last-tier treatments (*p* < 0.001, Table 1 and Fig. 1). The patients treated with DC had a higher rate of vegetative state/severe disability (65%) and a lower rate of mortality (25%) compared to those treated with thiopental, showing a vegetative state/severe disability rate of 21% and a mortality rate of 69%. Patients not requiring these treatments exhibited a vegetative state/severe disability rate of 34% and a mortality rate of 14%. For patients in the thiopental group who did not survive, most deaths (25/27 (93%)) occurred during NIC. Twenty-three of these 25 thiopental patients died due to severe intracranial hypertension when escalation to DC was considered counter-indicated due to the severity of the brain injury. The remaining two died later when the intracranial hypertension had ceased, but further care was withdrawn, and the patients were extubated due to pessimistic prognosis. No patient died as a complication to thiopental. When the long-term clinical outcome in patients surviving the NIC period was analyzed, thiopental patients showed 7% good recovery, 21% moderate disability, 57% vegetative state/severe disability, and 14% were dead at follow-up. In DC patients who survived NIC, 2% showed good recovery, 9% moderate disability, 76% vegetative state/severe disability, and 13% were dead at follow-up (Fig. 3).

The figure illustrates the relative GOS-E distribution for the patients who were not treated with last-tier ICP-treatments, those who were treated with thiopental alone, and those treated with DC surgery. The patients treated with DC had higher rate of vegetative state/severe disability (65%) and lower rate of mortality (25%) as compared to the patients treated with thiopental showing a vegetative state/severe disability rate of 21% and a mortality rate of 69%. Those who did not require these treatments exhibited a vegetative state/severe disability rate of 34% and had 14% mortality. In the thiopental group who did not survive, most deaths (25/27 (93%)) occurred during NIC. When the long-term clinical outcome in patients surviving the NIC period were analyzed, thiopental patients showed 7% good recovery, 21% moderate disability, 57% vegetative state/severe disability, and 14% were dead at follow-up. In DC patients who survived the NIC, 2% showed good recovery, 9% moderate disability, 76% vegetative state/severe disability, and 13% were dead at follow-up. Outcome definitions: Upper good recovery (full recovery, daily life not affected), lower good recovery (minor deficits that affect daily life), upper moderate recovery (some disability, partial resumption of previous work/daily life activities), lower moderate disability (independent, but no resumption of work/education/social activities), upper severe disability (partial assistance in daily life activities), lower severe disability (full assistance in daily life activities), vegetative state (no awareness), and dead (dead).

In the DC group, older age was associated with a lower GOS-E in Spearman correlation analyses (*r* =  − 0.44, *p* < 0.01), but WFNS grade, Hijdra score, DIND, or rebleeding were not associated with outcome. None of these variables were associated with GOS-E in the thiopental group. Older age (*r* =  − 0.32, *p* < 0.001), higher WFNS grade (*r* =  − 0.51, *p* < 0.001), and higher Hijdra score (*r* =  − 0.40, *p* < 0.001) correlated with a lower GOS-E in the cohort that did not receive thiopental or DC. In addition, those who had a rebleeding (median (IQR) 1 (1 − 4) vs. 5 (3 − 6), *p* < 0.001) and developed DIND (median (IQR) 4 (3 − 6) vs. 5 (3 − 6), *p* < 0.001) had a lower GOS-E at follow-up in the cohort that did not receive thiopental or DC.

## Discussion

In this study, one in 10 aSAH patients required last-tier treatments, including thiopental, DC, or both, to mitigate refractory intracranial hypertension. These patients were younger, in a worse neurological condition at admission, exhibited more blood on the initial CT scan, and more often suffered from rebleeding and DIND. Severe ICP problems were seen before these treatments were initiated, which were immediately alleviated after DC and to a lesser extent after thiopental. When thiopental patients surviving NIC were analyzed separately, we found a marked decrease in ICP. This finding illustrates a conscious selection where many patients receiving thiopental were assessed to have too poor prognosis to proceed with DC. Another striking observation was that only one in ten thiopental and DC patients achieved a favorable recovery overall, although a favorable outcome was seen in 28% of thiopental patients surviving NIC. This observation requires reflection and mandates for caution and careful patient selection when considering these last-tier treatments to relieve refractory intracranial hypertension in severely injured aSAH patients.

Of the 10% of patients who required any last-tier ICP treatment, 4% were treated with thiopental alone, 2% with thiopental followed by DC, and 4% with DC without prior thiopental. The DC rate at 6% in our study was slightly lower than 10%, which was the estimation in a recent meta-analysis by Darkwah Oppong et al. [8]. Some variation in these treatment rates is expected, given differences in patient cohorts on the extent of early brain injury, acute phase complications, and local ICP protocol. Few studies have investigated the benefits of thiopental in relieving intracranial hypertension in aSAH. It is therefore challenging to determine whether thiopental contributed to a lower rate of DCs in our cohort. However, in our population treated with thiopental but no DC, thiopental seemed sufficient to control ICP in at least the 14 patients who survived the NIC phase and did not need to proceed with the DC procedure.

Thiopental and DC patients showed a more severe primary brain injury, as indicated by a higher WFNS grade, a higher frequency of unreactive pupils, and a greater amount of blood on the initial CT. These patients also suffered more complications (e.g., rebleeding and DIND). In many cases, secondary and primary brain injury probably contributed to the refractory intracranial hypertension. The patients were also younger, which meant less brain atrophy and thus an even smaller additional volume could create ICP problems. Moreover, it probably reflects a lower threshold to proceed with aggressive treatments in younger patients because of a better chance of recovery.

Before thiopental and DC, the patients showed severe intracranial hypertension, as indicated by the radiological variables and the ICP monitoring data. Specifically, the thiopental patients showed signs of a global mass effect with compressed/obliterated cisterns and convexity sulci, but with limited midline shift. In contrast, DC patients also had a severe midline shift before treatment. These findings were consistent with our management protocol, i.e., not to use thiopental treatment but to proceed with DC if a significant midline shift has occurred. Thiopental was used in the progressive development of ICP situations when the patients were deemed treatable with relatively good chances and in situations when the likelihood of successful treatment was low but worth trying. Thiopental was also occasionally used to treat rapid ICP elevations directly following rebleeding. If there were a lack of ICP response combined with severe brain injury on imaging and the medical treatment was deemed futile, a decision was made to withdraw care rather than proceed with DC. This explains the high mortality rate (nearly two-thirds during NIC) in the thiopental cohort. This also explains why the variables of mass effect on imaging worsened, and ICP did not improve after thiopental in the entire cohort. Against this background, we analyzed surviving thiopental cases separately, and excluding fatal cases showed that ICP declined significantly as expected. It was also noteworthy that, although the favorable outcome rate was only 10% in the entire thiopental group, 28% of the survivors had a favorable neurological outcome. It should also be emphasized that in cases when thiopental did not relieve ICP and the brain injury was not considered too severe, a DC was performed to alleviate the intracranial hypertension.

DC patients differed slightly from those treated with thiopental alone and those who did not receive last-tier ICP treatments. The ruptured aneurysm was usually on the MCA and with an associated ICH, and thus several DC cases were treated with surgical clipping and simultaneous ICH evacuation. This treatment often resulted in secondary DC due to a combination of ICH-associated and cytotoxic edema from infarctions following clipping and DIND. DC led to immediate improvements in mass effect and ICP, which saved patients' lives, as the mortality rate was only slightly higher than the cohort without last-tier ICP treatments. Despite these findings, a favorable outcome was only reached in 10% of patients after DC, consistent with many studies on DC in aSAH [8, 14, 39, 42]. The most plausible explanation for this finding is the role of the underlying primary and secondary brain injury, which impeded functional recovery. However, even if a small proportion of DC patients had favorable outcomes, it is important to emphasize that 10% of the favorable outcomes after DC are relatively good when no other treatment options are available. Thus, based on this result, we suggest that DC in SAH should not be abandoned.

While DC was often life-saving, it created new problems. DC has been linked to the development of hydrocephalus, which was evident in our cohort as these patients more frequently required a VP-shunt [35, 43]. DC also necessitated CP surgery, which dictated further re-operations due to infections and bone resorption complications. In our study 42% of patients undergoing CP required a re-operation, which is relatively high compared to other studies [34, 40]. The high complication rate may reflect that autologous bone was mainly used, which often led to bone resorption [40] and that CP complications were assessed after a long follow-up time, up to 15 years for some CP patients treated in 2008, which allowed bone resorption to occur, be detected and treated. Using synthetic implants would probably have reduced the complication rate, since it obviates problems with bone resorption. Considering the challenges after DC, it appears preferable to use thiopental as the first choice in cases without focal mass lesions, given that many patients never needed DC and survived with favorable outcomes. However, if thiopental treatment does not reduce ICP in patients judged to have a reasonable chance of achieving a favorable outcome, management should be escalated to proceed with DC.

aSAH patients undergoing last-tier treatments seem worse than reported after thiopental and DC in TBI [24, 46] or DC in malignant media infarction [21, 29, 46]. The explanations for these discrepancies are not clear. Vasogenic edema is relatively common in TBI, contributing to ICP elevation but implies more viable tissue that can be saved by reducing intracranial hypertension than in cases with predominant cytotoxic edema [38]. In malignant media infarction, the brain edema is cytotoxic. However, the primary brain injury is regionally limited to the MCA territory, and the brain tissue outside this area is still viable if DC is done before further secondary brain injury occurs [9]. In aSAH, these patients are typically > 50 years old; the initial SAH inflicts a severe global insult to the entire brain rendering it vulnerable to secondary insults, and often ICP-problems reflect cytotoxic edema from infarction in a larger vascular territory. Accordingly, aSAH patients with ICP problems commonly exhibit an extensive initial global insult followed by a secondary focal brain injury, which may often be too severe to allow for functional recovery.

### Methodological considerations

The major strength of this study was the large study cohort, based on detailed data on clinical characteristics, the structured indications for last-tier ICP treatments, and radiological and ICP data before and after these treatments. This study adds to the evidence on the role of thiopental as an integral part of an escalated ICP protocol in aSAH. There were also some limitations as this was a retrospective, nonrandomized, single-center study without control groups for thiopental and DC patients, limiting external validity. In addition to differences in patient demographics and injury characteristics, variations in treatments, such as aneurysm occlusion (clipping/endovascular intervention), may influence the risk of secondary brain injury, development of ICP problems, and the necessity to proceed with last-tier treatments. Furthermore, in this study, slight developments in clinical care occurred throughout the study period, particularly regarding the technical advancements in endovascular interventions that might have changed the choice and success (rebleeding vs. complications) of the approach to treating the ruptured aneurysm [1]. Lastly, the radiological assessments were performed by only one of the authors (TSW, Associate Professor, neurosurgical resident), as it was very time-consuming, which may have decreased the accuracy, reliability, and interpretation of the results. However, it is unlikely that more than a few radiological examinations could have been misinterpreted and that the results would not be greatly affected. Therefore, it is reasonable to assume that the radiological assessments are sufficiently robust for the purposes of this manuscript.

## Conclusions

Refractory ICP elevations that did not respond to first-line treatments and required thiopental or DC surgery occurred in approximately 10% of the aSAH patients when a standardized treatment protocol was applied. The results show that DC effectively reduces ICP, is life-saving, and about 10% of patients may have a favorable outcome. However, many patients treated with DC live in a severe disability/vegetative state. When thiopental was used as the first-choice treatment in patients without mass lesions judged possible to treat and as an attempt in more fatal situations, favorable outcome was seen in 28% of patients surviving NIC, not including those where treatment was withdrawn or escalated to DC. This study shows that thiopental and DC offer important options as integrated last-tier treatment in aSAH. Still, careful patient selection based on a case-by-case approach is always paramount.

### Supplementary Information

Below is the link to the electronic supplementary material.

Supplementary file1 (DOCX 15 KB)

## Data Availability

Data is available upon reasonable request.
