# Supplementary material for: Thiopental and decompressive craniectomy as last-tier ICP-treatments in aneurysmal subarachnoid hemorrhage: is functional recovery within reach?
Source: Neurosurg Rev. 2023 Sep 7;46(1):231. doi: 10.1007/s10143-023-02138-6 (PMC10485091; doi:10.1007/s10143-023-02138-6)
Supplement: Supplementary file 1 — Supplementary file1 (DOCX 15 KB) [file 10143_2023_2138_MOESM1_ESM.docx]

**Supplementary table 1. Cranioplasty surgery and complications**

| DC patients operated with CP*, n (%) | 31 (60%) |
| --- | --- |
| Timing of CP post-DC (months), median (IQR) | 7 (4-9) |
| Type of implant (bone/synthetic), n (%) | 29/2 (94/6%) |
| Complications requiring re-operation | |
| Hematoma, n (%) | 0 (0%) |
| Infection, n (%) | 6 (19%) |
| Bone resorption, n (%) | 5 (16%) |
| Re-positioning, n (%) | 2 (6%) |
| Overall re-operations, n (%) | 13 (42%) |
| Timing of reoperation (months post-CP), median (IQR) | 8 (3-17) |

* Three were lost to follow-up as they were discharged to another neurosurgical facility, 1 patient did not want to proceed with CP, and the remaining 17 died before CP.

CP = Cranioplasty. DC = Decompressive craniectomy.
